# LRGUK-1 Is Required for Basal Body and Manchette Function during Spermatogenesis and Male Fertility

**DOI:** 10.1371/journal.pgen.1005090

**Published:** 2015-03-17

**Authors:** Yan Liu, Kathleen DeBoer, David M. de Kretser, Liza O’Donnell, Anne E. O’Connor, D. Jo Merriner, Hidenobu Okuda, Belinda Whittle, David A. Jans, Athina Efthymiadis, Robert I. McLachlan, Christopher J. Ormandy, Chris C. Goodnow, Duangporn Jamsai, Moira K. O’Bryan

**Affiliations:** 1 Department of Anatomy and Developmental Biology, School of Biomedical Sciences, Monash University, Australia; 2 MIMR-PHI Institute of Medical Research, Monash Medical Centre, Clayton, Australia; 3 Department of Urology, Osaka University Graduate School of Medicine, Osaka, Japan; 4 Australian Phenomics Facility, The Australian National University, Canberra, Australia; 5 Department of Biochemistry and Molecular Biology, Monash University, Clayton, Australia; 6 The Garvan Institute of Medical Research and St. Vincent’s Hospital Clinical School, UNSW Australia, Sydney, Australia; University of Nevada School of Medicine, UNITED STATES

## Abstract

Male infertility affects at least 5% of reproductive age males. The most common pathology is a complex presentation of decreased sperm output and abnormal sperm shape and motility referred to as oligoasthenoteratospermia (OAT). For the majority of OAT men a precise diagnosis cannot be provided. Here we demonstrate that leucine-rich repeats and guanylate kinase-domain containing isoform 1 (LRGUK-1) is required for multiple aspects of sperm assembly, including acrosome attachment, sperm head shaping and the initiation of the axoneme growth to form the core of the sperm tail. Specifically, LRGUK-1 is required for basal body attachment to the plasma membrane, the appropriate formation of the sub-distal appendages, the extension of axoneme microtubules and for microtubule movement and organisation within the manchette. Manchette dysfunction leads to abnormal sperm head shaping. Several of these functions may be achieved in association with the LRGUK-1 binding partner HOOK2. Collectively, these data establish LRGUK-1 as a major determinant of microtubule structure within the male germ line.

## Author Summary

Male infertility affects one in six couples in western societies and approximately half of these are the result of male factor disorders. The most common clinical presentation for male infertility is a complex mixture of abnormal sperm output, shape and motility referred to as oligoasthenoteratozoospermia (OAT). In an effort to define an origin of OAT we have analysed a mouse model of leucine-rich repeats and guanylate kinase-domain containing isoform 1 (LRGUK-1) dysfunction. Herein we show that LRGUK dynamically redistributes during the process of haploid germ cell maturation (spermiogenesis) and that LRGUK-1 function is required for multiple aspects of sperm centriole and tail development and sperm head shaping. Further, we have identified HOOK2 as a novel LRGUK-1 binding partner, thus raising the possibility that several aspects of LRGUK-1 function are achieved in partnership with HOOK2.

## Introduction

Male infertility affects at least 5% men of reproductive age in the western societies [1]. Normal male fertility requires sufficient numbers of morphologically normal and motile sperm [2]. Oligoasthenoteratozoospermia (OAT), is the term used to describe semen containing low numbers of sperm with poor motility and abnormal shape, and is the most common clinical phenotype in human male infertility [3]. The underlying aetiology in the majority of men presenting with OAT is unknown, and as such, there remains an absence of specific treatments, an absence of knowledge of associated somatic pathologies, and potential consequences for offspring conceived with OAT sperm via assisted reproductive technologies (ART).

The morphological and motility aspects of OAT likely have their origins in spermiogenesis, the process wherein round haploid germ cells are transformed into highly polarized sperm with the potential for motility and fertility. This process takes approximately two weeks in the mouse and involves several thousand different gene products [4]. Three of the major aspects of spermiogenesis are acrosome development, head shaping, and growth of the sperm flagellum [5,6]. These events are critically reliant upon complex microtubule structures, including the manchette and the axoneme, and a highly orchestrated series of protein transport mechanisms [7]. The manchette is a transient microtubule structure, which encircles the spermatid nucleus during the initial steps of elongation, and has a role in sculpting the species-specific nucleus shape. Abnormalities in manchette structure result in dysmorphic sperm heads [8–10]. We note that dynamic redistribution of the actin cytoskeletal system is also required for normal manchette function [8].

The axoneme forms the core of the sperm tail. It is composed of nine microtubule doublets surrounding a central microtubule pair (9+2) [11]. In contrast to motile cilia on other cells, the sperm tail axoneme is sheathed by accessory structures required for the production of ATP and protection against shear forces [12]. These structures include the outer dense fibers, fibrous sheath and mitochondrial sheath. Sperm tail axonemal development begins in round spermatids with the maturation of the mother centriole into a basal body, followed by plasma membrane attachment and axoneme extension. Several studies suggest that the extension of the basal body into an axoneme is dependent on the intra-flagella transport (IFT) system [13]. We note, however, that while this process is well defined in primary cilia [14], its role in sperm tail biology requires more in-depth analysis. Defectives in germ cell axoneme formation leads to either an absence of a sperm tail or immotile sperm.

Interestingly, research is increasingly indicating continuity between processes governing the development of the manchette and the sperm tail [13,15]. This may explain the extremely common association between abnormal sperm head morphology and motility in infertile men and mice [16]. Indeed, pioneering research from the Kierszenbaum lab has demonstrated a protein transport highway wherein proteins processed by the Golgi apparatus are transferred to the surface of the sperm acrosome at the cranial pole of the sperm head in a microtubule-dependent manner, then onto a cytoskeletal scaffolding plate known as the axoplaxome that anchors the developing acrosome to the nucleus [17]. Subsequently, proteins then localise to the microtubules of the manchette and ultimately into the growing sperm tail. The latter part of the highway has been termed intra-manchette transport and it appears to have mechanistic similarities with, and to interface with, the classical IFT pathway required for axoneme extension in most cilia types [13,18].

In an effort to understand these processes, we have undertaken a random mouse-mutagenesis screen to identify genes with critical roles in sperm formation. One of the genes identified encodes the previously uncharacterized gene leucine-rich repeats and guanylate kinase domain contain (*Lrguk*). Here we have shown that LRGUK isoform 1 (LRGUK-1) has a critical role in both sperm head shaping and basal body attachment to the plasma membrane, and in the early aspects of axoneme development. LRGUK is transported along the acrosome-acroplaxome-manchette-tail axis in a potential complex with the adaptor protein HOOK2 [19,20]. LRGUK-1 dysfunction leads to abnormal manchette formation and movement, and an absence of axoneme extension from the basal body. Collectively these data define LRGUK-1 as a crucial regulator of male germ cell basal body function, microtubule dynamics and fertility.

## Results

### *Lrguk* is required for male fertility

**Fig 1:**
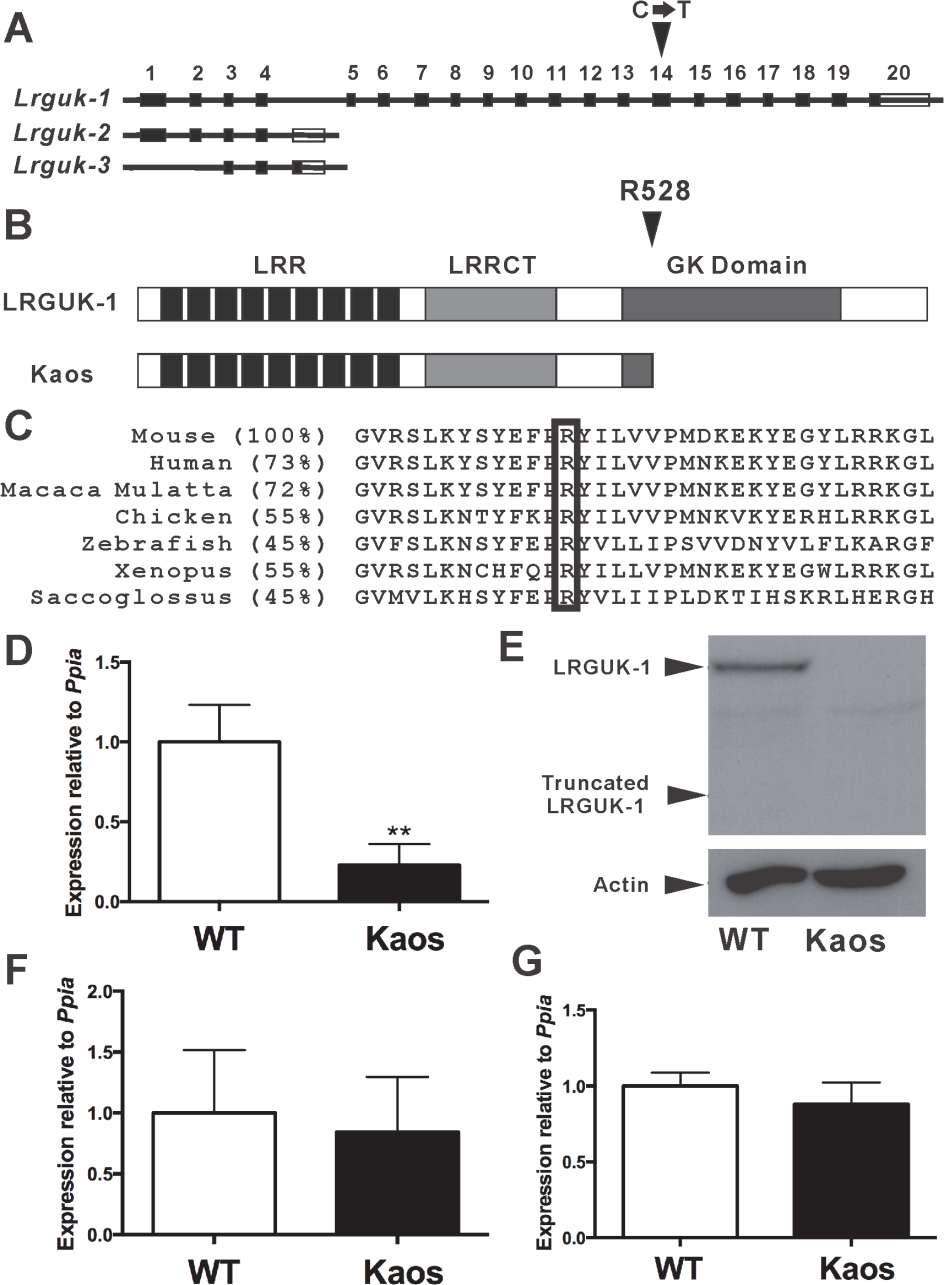
The Kaos mouse line phenotype was caused by a mutation in the *Lrguk* gene specifically affecting the LRGUK-1 isoform. **(A)** The *Lrguk* gene produces three transcripts in mouse. The Kaos mouse line contained a point mutation (C→T substitution, black arrowhead) located in exon 14 of *Lrguk* transcripts 1 (*Lrguk-1*). **(B)** The Kaos mutation resulted in the conversion of arginine 528 to a stop codon (R528*). LRR = leucine-rich region, LRRCT = leucine-rich repeat C-terminal domain, and GK = guanylate kinase-like domains. **(C)** A comparison of the relevant LRGUK region across species. The black box indicates the position of arginine 528. **(D)** *Lrguk* transcript 1 mRNA expression in *Lrguk* ^*Kaos/Kaos*^ (Kaos) versus wild type (WT) testes (n = 3 for each genotype). Expression was normalised against *Ppia* expression and shown as mean ± SEM. (**E**) LRGUK-1 content in *Lrguk* ^*Kaos/Kaos*^ (Kaos) versus wild type testes, 40μg of testis lysate per lane. Actin was used as a loading control. The predicted molecular weight of native LRGUK-1 was 93kDa and the predicted weight of the truncated LRGUK-1 protein was 60kDa. **(F)** A quantitative analysis of *Lrguk* transcript-2 and (**G**) *Lrguk* transcript-2 plus *Lrguk* transcript-3 in *Lrguk* ^*WT/WT*^ and *Lrguk* ^*Kaos/Kaos*^ testes (n = 3 for each genotype). Expression was normalized against *Ppia* expression and shown as mean ± SEM. ** denotes p<0.01.

In order to identify critical regulators of male germ cell development and fertility, we undertook a random N-ethyl-N-nitrosourea (ENU) mouse mutagenesis screen [9,21]. The ‘Kaos’ mouse line was identified based on male-specific infertility and the presentation of chaotic spermatogenesis (see below). Mapping of the mutation and sequencing of candidate genes revealed a C to T substitution in exon 14 of *Lrguk* (Fig. 1A). *Lrguk* is predicted to encode 3 splice variants, *Lrguk* transcript 1 *(Lrguk-1)*, -*2 (Lrguk-2)* and -3 *(Lrguk-3)*. *Lrguk-1* is the longest transcript and the only isoform affected by the mutation (Fig. 1A). The C→T mutation resulted in the conversion of an arginine (R) at position 528 to a premature termination codon (R528Stop, the *Lrguk* ^*Kaos*^ allele) and in turn the truncation of 293 residues from the C-terminal region of LRGUK-1 (Fig. 1B). Orthologues of LRGUK can be observed in many species, and sequence alignment of LRGUK-1 from multiple species revealed that R528 residue is conserved in all (Fig. 1C).

*Lrguk* ^*Kaos/Kaos*^ testis showed a 75% reduction in the level of *Lrguk*-*1* expression (8–12 weeks-of-age) compared to *Lrguk* ^*WT/WT*^ siblings (Fig. 1D) and a complete absence of both the native LRGUK-1 protein of 93 kDa and the predicted truncated LRGUK-1^Kaos^ of 60kDa (Fig. 1E). We note that the antibody used in these experiments is directed against an epitope present in both the native and truncated forms of LRGUK-1, but C-terminal to LRGUK-2 and -3 sequences. These results suggested *Lrguk-1* ^*Kaos*^ mRNA was unstable. Quantitative PCR (qPCR) showed no evidence of a compensatory up-regulation of *Lrguk-2* and *Lrguk-3* mRNAs in the *Lrguk* ^*Kaos/Kaos*^ testis (Fig. 1F-G). Collectively, these data revealed that *Lrguk* ^*Kaos/Kaos*^ males were sterile as a consequence of a specific absence of the LRGUK-1 protein.

### *Lrguk* ^*Kaos/Kaos*^ males sterility was characterised by oligoasthenoteratospermia

**Fig 2:**
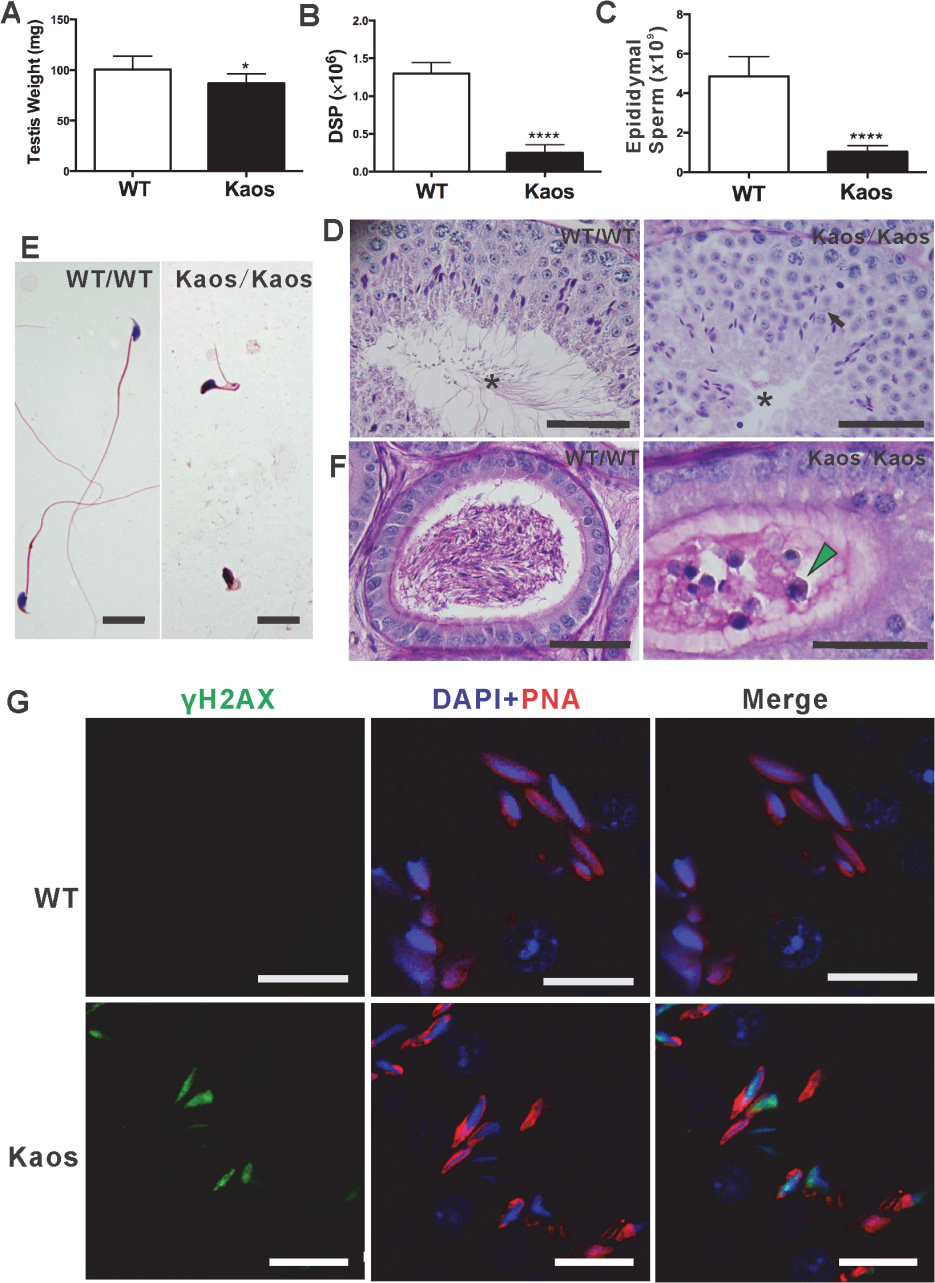
*Lrguk* ^*Kaos/Kaos*^ males are oligoasthenoteratospermic and sterile. **(A)** A comparison of testis weight from *Lrguk* ^*WT/WT*^ (WT) and *Lrguk* ^*Kaos/Kaos*^ (Kaos) adult mice (age = 8–12, n = 12–13 per genotype +/- SEM). **(B)** Daily sperm production (DSP) by the testes from *Lrguk* ^*WT/WT*^ and *Lrguk* ^*Kaos/Kaos*^ adult mice (n = 7 per genotype +/- SEM). **(C)** Total epididymal sperm content from *Lrguk* ^*WT/WT*^ and *Lrguk* ^*Kaos/Kaos*^ adult mice (n = 7 per genotype). (**D**) PAS stained testis sections from *Lrguk* ^*WT/WT*^ and *Lrguk* ^*Kaos/*^ mice. * denotes the absence of sperm tails in the lumen of *Lrguk* ^*Kaos/Kaos*^ males; black arrowheads point to fragmented acrosomes. Scale bar = 50μm. **(E)** Haematoxylin and eosin stained sperm smears from *Lrguk* ^*WT/WT*^ and *Lrguk* ^*Kaos/Kaos*^ animals. Scale bar = 10μm. **(F)** Haematoxylin and eosin staining of an epididymis from *Lrguk* ^*WT/WT*^ and *Lrguk* ^*Kaos/Kaos*^ mice. The green arrow indicates sloughed of immature germ cells. Scale bar = 50μm. **(G)** γH2AX foci (green) were visible in retained step I-III elongated spermatids from *Lrguk* ^*Kaos/Kaos*^ mice but not *Lrguk* ^*WT/WT*^ spermatids. Acrosomes were visualised by PNA (red) and nuclei were visualised with Dapi (blue). Scale bars = 10 μm. *p<0.05, ****p<0.0001.

All *Lrguk* ^*Kaos/Kaos*^ males had chaotic and disorganised spermatogenesis and were sterile (n>50, aged 7–26 weeks), whereas *Lrguk* ^*WT/Kaos*^ males and *Lrguk* ^*Kaos/Kaos*^ females had apparently normal fertility. *Lrguk* ^*Kaos/Kaos*^ males had a normal body weight (S1A Fig.) and were anatomically indistinguishable from wild type siblings. *Lrguk* ^*Kaos/Kaos*^ male sterility was characterized by a 13% reduction in testis weight (Fig. 2A) and an 81% reduction in daily sperm production (Fig. 2B). Collectively these data suggest that the majority of missing germ cells were from the haploid phase of spermatogenesis wherein they contributed relatively little to testis weight. A similar reduction (79%) in sperm content was observed in the epididymal sperm content (Fig. 2C). The loss of germ cells from the seminiferous epithelium via sloughing was indicated by the presence of immature germ cells in *Lrguk* ^*Kaos/Kaos*^ epididymis (Fig. 2F). In contrast, apoptosis levels were similar between genotypes (S1B-C Fig.). Of the elongate spermatids present in the seminiferous epithelium, virtually none appeared to contain a sperm tail (Fig. 2E), and of the very few sperm that were present in the epididymis, all had grossly misshapen heads and shortened tails (Fig. 2D) that displayed no capacity for motility. A closer examination of spermatid structure also revealed the presence of fragmented acrosome in early round spermatids (Fig. 2E and below).

A more quantitative stereological analysis of the dynamics of spermatogenesis confirmed the histological observations. *Lrguk* ^*Kaos/Kaos*^ adult testes contained normal numbers of supporting Sertoli cells, spermatocytes and early round spermatids (S1 Table). They contained, however, a 37% reduction (p<0.05) in elongated spermatids (steps 13–16). In addition, we noted a pronounced increase in the number of elongated spermatids retained in the basal crypts of Sertoli cells in stage VII-XII tubules that was suggestive of a failure of the early phases of sperm release (spermiation)(S1D Fig.). Consistent with this interpretation, the levels of DNA double strand breaks, as determined by γH2AX staining, in the retained spermatid nuclei was notably elevated (Fig. 2G) in *Lrguk* ^*Kaos/Kaos*^ suggesting the initiation of degeneration [22].

Cumulatively, these data show that *Lrguk* ^*Kaos/Kaos*^ males were sterile as a consequence of germ cell sloughing and degeneration, abnormal sperm development and an inability of those sperm that were produced to ascend the female reproductive tract following mating.

### LRGUK-1 is testis-enriched and associated with the acrosome-acroplaxome-manchette-tail axis

**Fig 3:**
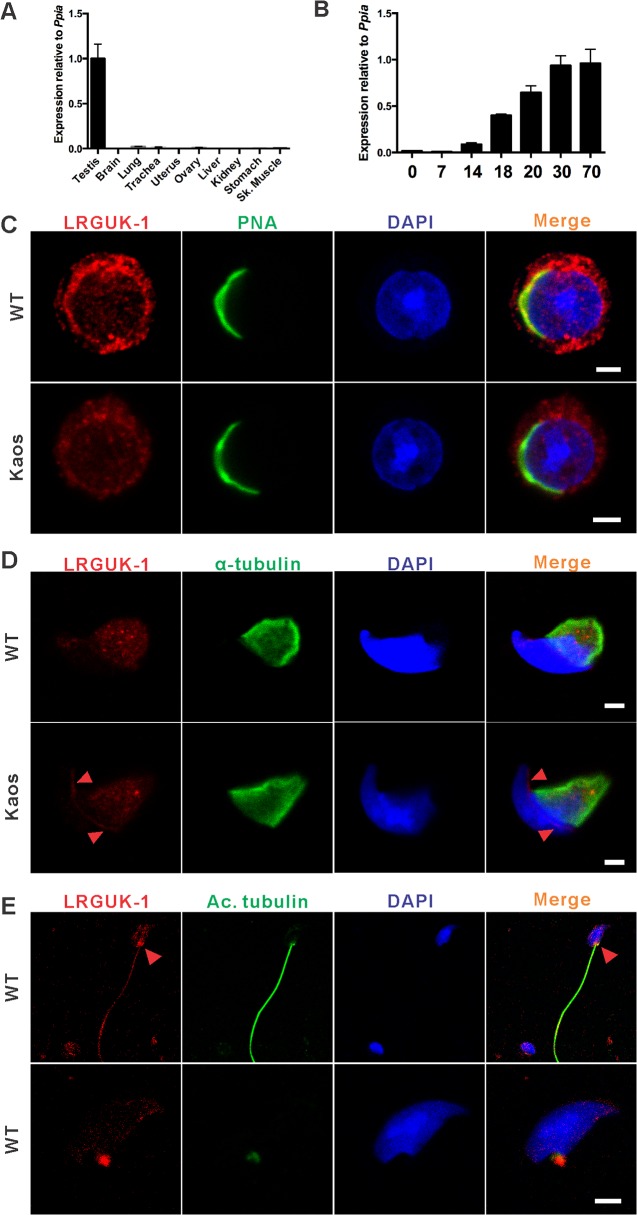
LRGUK-1 is highly expressed in the testis and the localizes to the acrosome-acroplaxoneme-manchette-tail network. (**A**) Quantitative PCR analysis of *Lrguk-1* mRNA expression in various tissues **(B)** and during the establishment of the first wave of spermatogenesis n = 3. Expression was normalised against *Ppia* expression and shown as mean±SEM. (**C**) The localization of LRGUK (red) in purified round spermatids, scale bar = 2μm. Acrosomes were visualised by PNA (green). **(D)** The localization of LRGUK-1 (red) in purified elongating spermatids, co-labelled with α-tubulin (green) to mark the manchette (scale bar = 2 μm). The red arrowhead indicates LRGUK localization at the leading edge of the acrosome **(E)** The localization of LRGUK (red) within caudal epididymal sperm, co-labelled with acetylated tubulin (Ac, green) to mark the axoneme (upper panel) and sperm basal body (upper and lower panel). Red arrowheads denote LRGUK localization in the sperm basal body. Scale bar = 4 μm. In all images, nuclei were labelled with DAPI (blue). Please see S3 Fig. for negative control images.

qPCR analysis revealed that *Lrguk-1* mRNA was highly enriched in the testis compared to other adult tissues (Fig. 3A). An analysis of mouse testes taken at defined periods during the establishment of the first wave of spermatogenesis also revealed that *Lrguk-1* mRNA was detectable at low levels from birth, up-regulated at day 14 coincident with the appearance of pachytene spermatocytes, then maximal from day 18 coincident with the appearance of haploid germ cells (Fig. 3B). This result was suggestive of *Lrguk-1* being predominantly expressed in haploid germ cells. During spermatid development, and using an antibody that should detect all of LRGUK1–3, LRGUK protein was initially localised to a supra-nuclear region of round spermatids, and was particularly evident at the leading edge of the developing acrosome and acroplaxome (Fig. 3C). As maturation proceeded and nuclear elongation initiated, LRGUK moved distally to ultimately reside on the microtubules of the manchette (Fig. 3D and S2 Fig.). LRGUK was also evident in the sperm basal body and the sperm tail (Fig. 3E). These data, and the abnormal sperm head and tail morphology in *Lrguk* ^*Kaos/Kaos*^ germ cells, suggested that LRGUK-1 has a role in acrosome and tail biogenesis. We note that LRGUK protein was not detectable in our hands in spermatocytes. At present it is unknown if this was due to a lack of abundance or a translational delay as is often seen in spermiogenic genes [23].

*Lrguk* ^*Kaos/Kaos*^ germ cells showed reduced LRGUK immunolabelling compared to wild type cells (S2 Fig.), however, residual, presumably LRGUK-2 and/or LRGUK-3 staining could be seen within mutant spermatids (Fig. 3C-D and S2 Fig.). The possibility also remains that a small amount of truncated LRGUK-1 was detected by this antibody. Within these cells the distribution of residual LRGUK was notably perturbed. Specifically, the movement of LRGUK appeared to stall at the leading edge of the acrosome/acroplaxome complex (Fig. 3C-D and S2 Fig.). LRGUK staining on the microtubules of the *Lrguk* ^*Kaos/Kaos*^ manchette was reduced and more variably distributed (Fig. 3D and S2 Fig.). These data are consistent with the acroplaxome operating as a loading dock for cargo proteins prior to loading onto the microtubules of the manchette [15,17] and the C-terminal 293 amino acids of LRGUK-1 having a role in this transition.

### LRGUK is involved in sperm head formation

**Fig 4:**
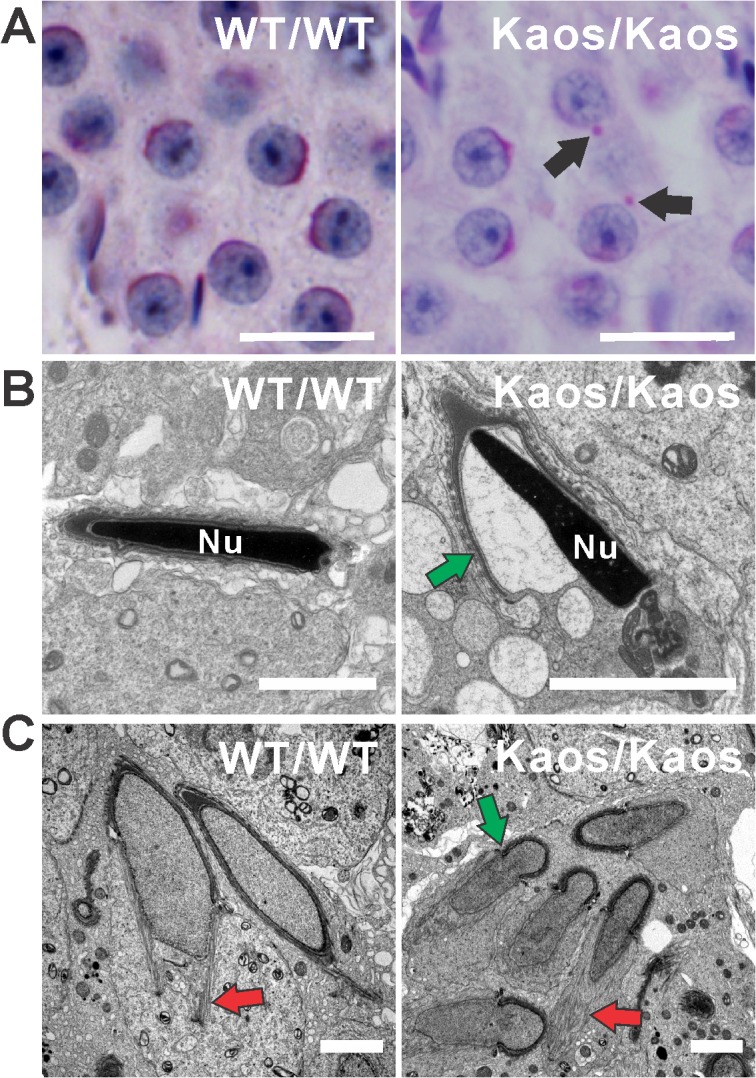
Acrosome and manchette defects in *Lrguk* ^*Kaos/Kaos*^ mice. **(A)** PAS stained testis sections from *Lrguk* ^*WT/WT*^ and *Lrguk* ^*Kaos/Kaos*^ mice. Arrows indicate mis-localised proacrosomal vesicles. **(B)** Transmission electron microscopy of elongated spermatid from *Lrguk* ^*WT/WT*^ and *Lrguk* ^*Kaos/Kaos*^ mice. The green arrow indicates a detached acrosome. Nu = nucleus. Scale bar = 2μm. **(C)** Transmission electron microscopy showing abnormal manchette structure including a constricted perinuclear ring (green arrow). Red arrows indicate microtubules within the manchette.

One of the features of the *Lrguk* ^*Kaos/Kaos*^ phenotype was the abnormal morphology of sperm heads. A detailed analysis of acrosome formation on periodic acid Schiff’s (PAS) stained sections revealed the presence of fragmented acrosomes in step 2–4 spermatids (Fig. 4A). The acrosome is formed by the sequential fusion of Golgi-derived pro-acrosomal vesicles to form a cap overlying the nucleus. The membrane overlying the acrosome is essential for the initial binding to and penetration through the oocyte complex [15,24,25]. Electron microscopy also revealed ∼20% of acrosomes in elongated spermatids were detached from sperm nuclei (Fig. 4B). A close inspection of these cells indicated that both the acrosome and the acroplaxome were detaching from the nuclear membrane suggesting that LRGUK-1 has a role in establishing the integrity of the connection between the acroplaxome and the nuclear membrane.

**Fig 5:**
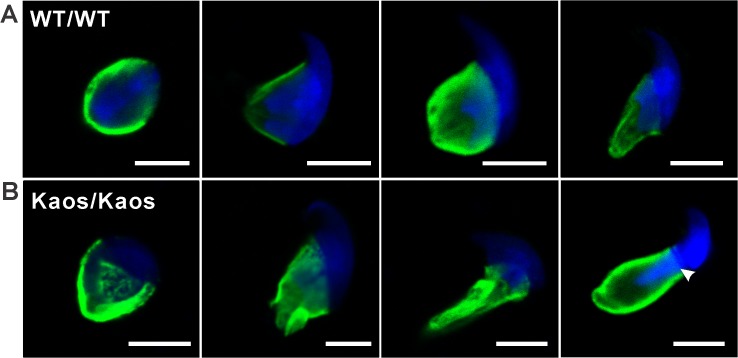
Manchette structure in *Lrguk* ^*WT/WT*^ and *Lrguk* ^*Kaos/Kaos*^ elongating spermatids. α-tubulin staining (green) of manchette microtubules in elongating spermatids from *Lrguk* ^*WT/WT*^ **(A)** and *Lrguk* ^*Kaos/Kaos*^ mice **(B)** are shown in progressive steps during head elongation. Nuclei were stained with DAPI (blue). The scale bar = 5 μm. Please see S3 Fig. for negative control images.

*Lrguk* ^*Kaos/Kaos*^ spermatids also contained abnormal manchettes (S2 Fig., Fig. 4D and Fig. 5). The manchette is a grass skirt-like structure that encircles the elongating spermatid nucleus. As indicated above, it has a role in both nuclear shaping and protein transport into the growing sperm tail via a process called intra-manchette transport [13,18]. In normal spermatids, the manchette contains a series of parallel microtubule bundles that extend from a perinuclear ring and lie in close proximity to, and are parallel with, the nuclear membrane into the distal cytoplasm (Fig. 4C and Fig. 5). An analysis of elongating *Lrguk* ^*Kaos/Kaos*^ spermatids, using α-tubulin as a microtubule marker, revealed that the manchettes formed at the correct time and that the perinuclear ring began to move caudally along the sperm head during spermiogenesis (Fig. 5). The microtubule bundles of the manchette skirt were, however, unevenly distributed and had a ‘raggedy’ appearance compared to wild type cells. Further, and based on both the light and electron microscopic images the distal movement of the manchette along the nucleus that normally occurs during elongation was also abnormal in *Lrguk* ^*Kaos/Kaos*^ spermatids (Fig. 5, S3 Fig.). In contrast the perinuclear ring of the manchette continued to constrict, as it would normally, thus leading to nuclear distortion (Fig. 4C, Fig. 5 and S2 Fig.).

Collectively these data illustrate a role for LRGUK-1 in acrosome attachment to the sperm head and in microtubule organisation within the manchette.

### LRGUK binds to HOOK2 and functions in sperm tail axoneme extension

**Fig 6:**
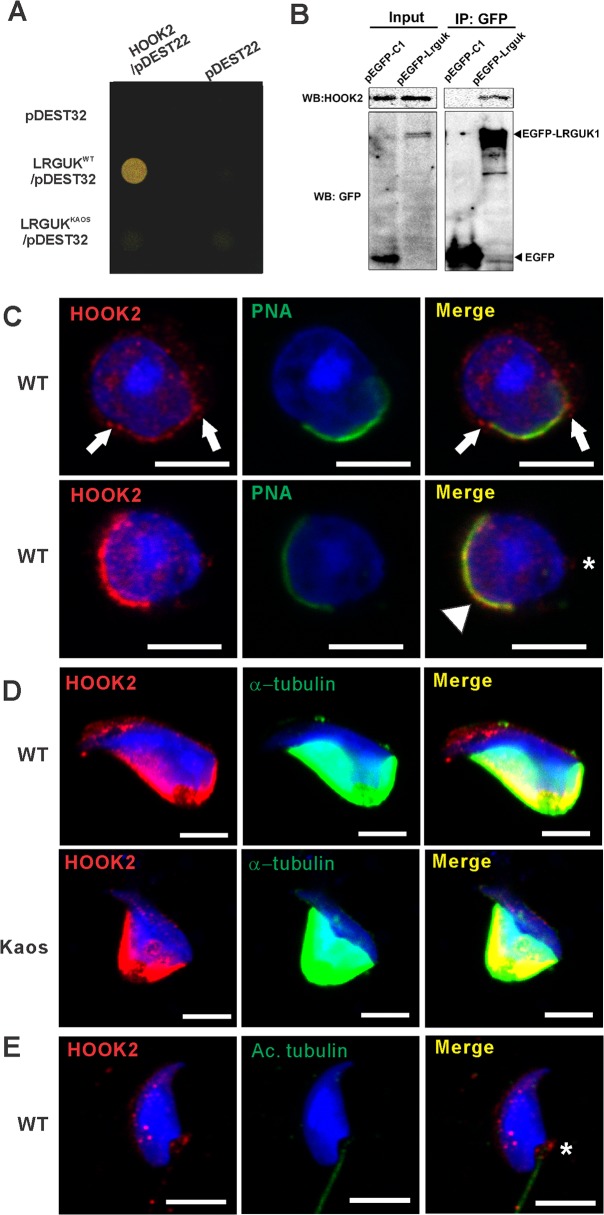
LRGUK-1 binds to HOOK2. **(A)** Growth of yeast carrying wild type LRGUK-1 (LRGUK^WT^/pDEST32) and HOOK2 (HOOK2/pDEST22) compared to those carrying LRGUK^Kaos^ (LRGUK^Kaos^/pDEST32) and HOOK2 (HOOK2/pDEST22), and empty pDEST32 and pDEST22 vectors. Binding was absent (i.e. no growth) when empty vectors were used as a negative control. (**B**) Confirmation of the interaction between LRGUK-1 and HOOK2 by immunoprecipitation from HEK293 cells expressing EGFP-tagged LRGUK-1. EGFP-tagged LRGUK-1 was immunoprecipitated using GFP antibody-conjugated beads (anti-GFP-Trap-A beads), and immunoblotting was performed using HOOK2 antibody to detect the interaction between the EGFP-tagged LRGUK-1 and endogenous HOOK2. pEGFP-C1 represents a negative control which over-expressed EGFP protein only. Input: whole cell lysates of transfected HEK293 cells. **(C)** The localization of HOOK2 (red) in WT round spermatids, co-labelled with PNA (green) to mark the acrosome. HOOK2 was present within the developing acrosome/acroplaxome (arrowhead), at a region consistent with the leading edge of the acrosome and acroplaxome (arrows) and at a site consistent with the basal body (asterisk). **(D)** The localisation of HOOK2 (red) onto the microtubules (α-tubulin, green) of the manchette in WT and *Lrguk* ^*Kaos/Kaos*^ elongating spermatids. Scale bar = 5 μm. (**E**) HOOK2 (red) within caudal epididymal sperm. Acetylated (Ac) tubulin was used as an axoneme marker. The asterisk indicates HOOK2 in the sperm basal body. Scale bar = 5 μm. In all images, nuclei are labelled with DAPI (blue). Please see S3 Fig. for negative control images.

LRGUK-1 contains multiple domains with known roles in protein-protein interactions i.e. a guanylate kinase-like domain (GK), leucine rich repeats (LRRs) domains and a leucine rich repeat C-terminal domain (LRRCT) [26,27] (SMART entry IPR000483). In order to explore this potential, and to define pathways within which LRGUK-1 may be involved during haploid germ cell development, we performed a yeast two-hybrid screen to identify LRGUK-1 binding partners. One of the binding partners identified was HOOK2 (Fig. 6). The identified *Hook2* clone encoded the C-terminal 348 amino acids of HOOK2. Specific transfection of the full length HOOK2 with LRGUK-1 in a separate yeast two hybid assay confirmed this interaction and the introduction of the R528Stop mutation into the *Lrguk-1* sequence completely abolished binding to HOOK2 (Fig. 6A). An interaction between LRGUK-1 and endogenous HOOK2 was confirmed by co-immunoprecipitation, wherein full-length mouse *Lrguk-1-GFP* was transfected into HEK293T cells then the complex co-precipitated (Fig. 6B).

HOOK2 is a member of the HOOK family of proteins, which are adaptor-like proteins involved in loading cargos (including protein complexes and organelles) onto microtubules for transport [28]. Notably, HOOK2 has recently been found to function in the maintenance of centriole structure and primary cilia assembly [19]. Like sperm flagella, primary cilia contain a central axoneme, but rather than possessing a 9+2 microtubule structure, they lack the central microtubule pair (9+0) [16,29]. Specifically, within retinal epithelial cells HOOK2 bound to the essential cilia proteins PCMI and RAB8a and was required to initiate axoneme growth from the basal body [20]. These data raise the possibility that HOOK2 is involved in the delivery of LRGUK-1 to the basal body, or *vice versa*, and in the initiation of sperm tail growth.

As for LRGUK, HOOK2 was localised to the Golgi-derived spermatid acrosome, the acroplaxome, the manchette and the sperm basal body (Fig. 6C-E). HOOK2 localization was not appreciably altered in the presence of the *Lrguk* ^*Kaos*^ mutation, *albeit* in the presence of abnormal germ cell structure (Fig. 6D), indicating that LRGUK-1 does not define HOOK2 localization. Unfortunately genetically modified *Hook2* mouse lines are not available to test the dependence of LRGUK localization on HOOK2. As such, and although the formal possibility remains that HOOK2 may be a LRGUK-1 cargo, it is more likely that HOOK2 functions in its established role to transport LRGUK-1.

**Fig 7:**
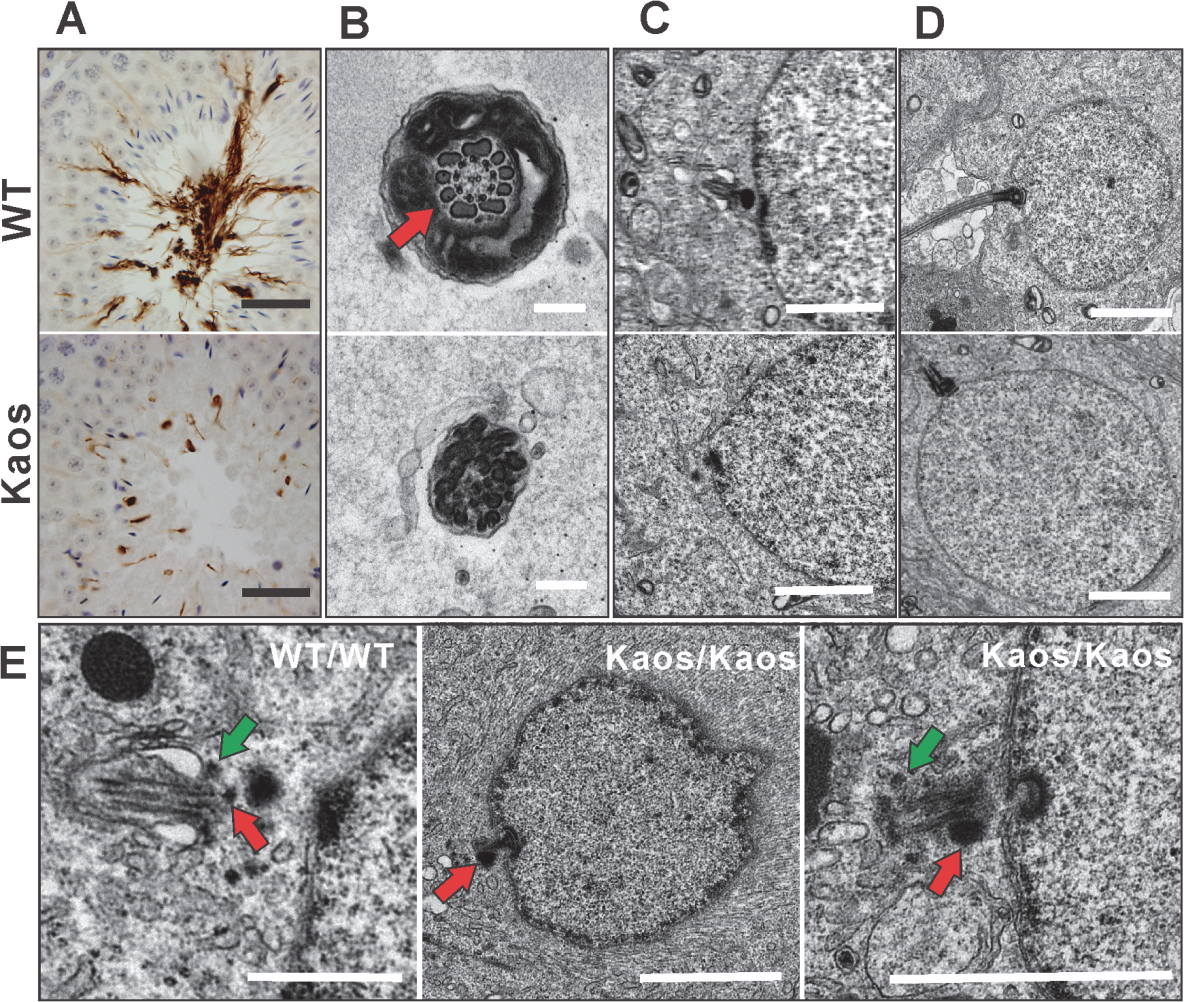
Axoneme defects in *Lrguk* ^*Kaos/Kaos*^ mice. **(A)** The staining of testis sections with the axoneme marker, acetylated tubulin, indicated an almost complete absence of axoneme development in *Lrguk* ^*Kaos/Kaos*^ germ cells. **(B)** Transmission electron microscopy indicating the absence of axoneme development in wild type and *Lrguk* ^*Kaos/Kaos*^ elongating spermatids. Red arrows indicate outer dense fibers. Scale bar = 0.2 μm. (**C-D**) Transmission electron microscopy of basal body docking to the plasma and nuclear membrane (C) and subsequent axoneme extension in *Lrguk* ^*WT/WT*^ cells but the absence of plasma membrane attachment and axoneme of extension in *Lrguk* ^*Kaos/Kaos*^ spermatids. Scale bar = 2 μm. (**E**) A closer examination of the early phases of basal body structure and movement revealed the presence of normal distal appendages (DAPs, green arrow)) and sub-distal appendages (SAPs, red arrow) in wild type cells associated with plasma membrane attachment. *Lrguk* ^*Kaos/Kaos*^ germ cells contained enlarged SAP and the basal body failed to associate with the plasma membrane. The scale bar = 2μm.

The *Lrguk* ^*Kaos/Kaos*^ sperm phenotype has some similarities with that observed for HOOK2 depleted retinal cells, suggesting they lie in a common pathway. Greatly reduced axoneme development was evidenced in testis sections stained for the axoneme marker acetylated tubulin and the absence of 9+2 axoneme structure was seen at the electron microscopic level (Fig. 7A-E). An analysis of the early steps of centriole/basal body movement and axoneme development, however, revealed abnormalities in *Lrguk* ^*Kaos/Kaos*^ germ cells, strongly suggestive of a critical role for LRGUK-1 beyond that currently documented for HOOK2, specifically in the formation of the centriole appendages. In early wild type spermatids the mature centriole can be distinguished from the adjacent daughter centriole by the possession of accessory structures known as the sub-distal appendages (SAP) and distal appendages (DAPs) [30]. The mature centriole then migrates to the spermatid periphery and attaches to the plasma membrane before attaching to the nuclear pole opposite the acrosome [16]. Recent data has shown the DAPs are required for basal body-to-membrane docking [31], and SAPs are believed to be where axoneme microtubules are anchored and are thus required for axoneme extension [32–34]. Electron microscopy of spermatids from *Lrguk* ^*WT/WT*^ spermatids clearly showed the docking of mature centrioles to the plasma membrane and the associated DAPs and SAPs (Fig. 7E). In contrast, the over-whelming majority of basal bodies in spermatids from *Lrguk* ^*Kaos/Kaos*^ mice contained SAPs that were overtly enlarged and plasma membrane attachments were very infrequent (<1% of the time) (Fig. 7E). Consistent with abnormal SAP function, microtubule extension from the *Lrguk* ^*Kaos/Kaos*^ basal body into an axoneme was extremely rare (Fig. 7D). Despite the absence of membrane attachment basal bodies did contain DAP-like structures. *Lrguk* ^*Kaos/Kaos*^ basal bodies did, however, appeared to attach to the nuclear membrane normally (Fig. 7E). In comparison to the basal body phenotype seen in *Lrguk* ^*Kaos/Kaos*^ spermatids, the loss of HOOK2 function during retinal cell primary cilia formation had no reported effect on SAP formation [19]. These data suggest that LRGUK-1 is required for the formation / function of the DAPs and SAPs in spermatids. Currently it is unknown if these effects are independent of HOOK2 or if they are spermatid-specific functions and thus, not seen following HOOK2 knockdown in retinal cells.

In contrast to the lack of axoneme development in *Lrguk* ^*Kaos/Kaos*^ spermatids, we observed considerable evidence of the assembly of outer dense fiber-like structures within the distal cytoplasm of elongated spermatids (Fig. 7B). These data suggest that while the outer dense fibers sit in close apposition to the microtubules of the axoneme in normal germs cells, their development can occur independently.

## Discussion

The dynamic organisation of microtubules and the ability to transport proteins over long distances are fundamental processes required for many cell types, but perhaps none more so than haploid male germ cells, where sperm head shaping and tail development occurs in the virtual absence of transcription [35]. Here we have demonstrated that LRGUK-1 is a critical component of this process with roles that impact upon multiple facets of sperm structure. LRGUK-1 is required for normal acrosome attachment, manchette function, the initiation of the axoneme extension and ultimately male fertility. In accordance with these roles, we observed LRGUK-1 within a protein transport corridor [13] involving movement from the Golgi complex in round spermatids, to the acrosome/acroplaxome, onto the manchette in elongating spermatids then ultimately into the sperm tail. Collectively our data establish LRGUK-1 as a vital component for haploid germ cell development and male fertility.

The earliest discernable abnormality seen in *Lrguk* ^*Kaos/Kaos*^ germ cells was in acrosome development. The acrosome is a vesicle-like structure at the caudal pole of the sperm head. The plasma membrane immediately above the acrosome is the first point of binding between the sperm and the cumulus oocyte complex prior to fertilisation, and the contents of the acrosome is required for sperm penetration through the outer vestments of the oocyte [36]. Data presented here suggests that LRGUK-1 is required for the appropriate attachment of Golgi-derived pro-acrosomal vesicles onto the nucleus and the full structural integrity of the acrosome-acroplaxome attachment to the nuclear membrane [15,17]. LRGUK-1 dysfunction resulted in the fragmentation and loss of some pro-acrosomal vesicles in early round spermatids, and the detachment of the acrosome-acroplaxome from the sperm head during late spermiogenesis. Clearly however, acrosome-like structures were formed in spermatids (*albeit* with apparently reduced efficiency in early round spermatids) indicating that the enhanced adhesion inferred by LRGUK-1 may only become critical when shear forces are applied, for example during stages I through to VIII wherein elongated spermatids are sequentially dragged down into Sertoli cell crypts then pushed up to the luminal aspect of the seminiferous epithelium [37]. The apparent absence of LRGUK-1 protein from the acroplaxome region in mature sperm suggests that this defect maybe mediated by an LRGUK-1 binding partner rather than LRGUK-1 itself, or that LRGUK-1 is part of a stepwise process resulting in firm acrosome-acroplaxome-nuclear attachment. Taken together our results suggest a role for LRGUK-1 in the trafficking of pro-acrosomal vesicles from the Golgi to the acroplaxome and in the attachment of acrosome-acroplaxome to the nuclear membrane.

A second major defect, and almost certainly the cause of the head abnormalities in *Lrguk* ^*Kaos/Kaos*^ sperm, was a defect in the structure and movement of the manchette. The manchette is a complex microtubule array that forms around the spermatid nucleus concordant with the initiation of nuclear compaction. It is composed of a perinuclear ring and a fringe of microtubule bundles that extend into the distal cytoplasm. As spermiogenesis proceeds the manchette moves distally, and in the case of species such as the mouse that contain falciform shaped sperm heads, it pivots in a manner dependent on microtubule severing [22]. In parallel, the perinuclear ring constricts, likely contributing to the tapered shape of the post-acrosomal region of the sperm head. Our data show that LRGUK-1 is required for the distal movement of the perinuclear ring. In the presence of the *Lrguk* ^*Kaos/Kaos*^ mutation the perinuclear ring constricted more proximally than normal leading to abnormal sperm head shape. Interestingly the remaining LRGUK present within elongating spermatids (LRGUK-2 and/or -3 or small amounts of truncated LRGUK-1) remained at the leading edge of the acroplaxome, suggesting that there are motifs C-terminal 293 amino acids of LRGUK-1 that are required for the transition to the manchette.

Of relevance, a second member of the HOOK family is required for manchette function and ultimately male fertility. The *azh* mouse phenotype was caused by the deletion of exons 10–11 from the *Hook1* gene [38]. Homozygous *azh* males displayed OAT characterised by severe head abnormalities and the frequent decapitation from the sperm tail. The latter is suggestive of a weakened connection between the nuclear membrane and the basal body-derived axoneme. This phenocopying and data suggesting that HOOK family members frequently function as heterodimers [39] raise the possibility that LRGUK-1 exists in a complex with both HOOK2 and HOOK1. This possibility will be tested in future experiments.

The most striking aspect of the Kaos phenotype, and the ultimate cause of male sterility, was the almost complete block of sperm tail development. LRGUK-1 dysfunction lead to abnormal SAP formation on basal bodies and an absence of plasma membrane attachment, likely associated with DAP dysfunction. As a consequence, basal bodies failed to nucleate axoneme microtubules. Within retinal primary cilia, HOOK2 is involved in the assembly of a complex containing PCMI and RAB8a, and the initiation of axoneme microtubule extension [19]. In contrast to HOOK2 action in primary cilia, however, our data revealed that LRGUK-1 also has a critical role in the formation of basal body-plasma membrane attachment and SAP formation. Currently it is unknown if these effects are independent of HOOK2 or if they are spermatid-specific functions and thus, not seen following HOOK2 knockdown in retinal cells. Our data do not, however, support a role for LRGUK-1 in cilia broadly. Notably, the Kaos mouse line showed none of the morphometric features characteristic of primary cilia disorders such as polydactyly and craniofacial abnormalities [40], although the presence of *Lrguk-1* mRNA in tissues containing motile cilia (9+2 axonemes) raises the possibility of an age-related pathology associated with motile cilia dysfunction.

The phenotypic similarity observed in *Lrguk* ^*Kaos/Kaos*^ and mice containing mutations in core proteins of the IFT pathway including *Ift88* and *Kif3a* [41,42] highlights the continuity of the protein transport pathway underlying the movement of proteins involved in sperm head shaping and axoneme extension. Our data suggest that LRGUK-1 functions upstream/before the IFT pathway i.e. spermatids with defective LRGUK-1 will undergo a developmental arrest prior to the requirement for the IFT pathway. These data also highlight the exquisite value of using the testis as a model to define processes of likely fundamental importance to cell biology broadly.

Collectively, we have identified LRGUK-1 as a protein critically involved in multiple aspects of sperm assembly and function. LRGUK-1 is required for appropriate acrosome attachment to the sperm heard, sperm head shaping via the manchette and tail growth from the basal body. During the initiation of sperm tail axoneme extension from the basal body, LRGUK-1 functions in plasma membrane attachment and the formation of SAP and ultimately microtubule extension. The specific loss of LRGUK-1 function results in OAT in mice and raises the possibility that *LRGUK* dysfunction leads to human OAT.

## Materials and Methods

### Identification of the Kaos line and the causal mutation

Animal procedures were performed in accordance with Australian NHMRC Guidelines on Ethics in Animal Experimentation and approved by the Australian National University and Monash University Animal Experimentation Ethics Committees. Point mutant mice were generated on a C57BL/6 background and outbred to CBA and individual lines screened for sterility causing mutations as described previously [9,21]. Mating behaviour, as indicated by the presence of copulatory plugs was normal.

The sterility causing mutation in the Kaos line was initially linked to a region on chromosome 6 using a SNP-based microarray approach. The region was subsequently narrowed using additional mice and SNPs to a 12.9 Mb region (30872499–43776812 bp) that contained 74 genes. Candidate genes were selected based on expression in haploid germ cells, i.e. the site of the phenotype, in EST expression databases. 28 genes were expressed within round spermatids. The full coding region of eight of these genes was sequenced and a single C to T point mutation within the *Lrguk* gene was identified.

Following the identification of the causal mutation, mice were genotyped using the Amplifluor system (Chemicon) using a wild type-specific reverse primer 5’-GAAGGTCGGAGTCAACGGATTCCATAGGCACCACCAAGATATATCG-3’, a mutant allele specific reverse primer 5’-GAAGGTGACCAAGTTCATGCTCCA TAGGCACCACCAAGATATATCA-3’ and a conserved forward primer 5’- CAGCCTTGGACTATTTATAGGGAGTGTG-3’ as described previously [9].

LRGUK orthologues were identified as described previously [7].

### Characterization of the Kaos sterility phenotype

Sterility in the Kaos mouse line was characterized using the regime outlined in Borg et al [43]. Daily sperm production (DSP) in the testis and total epididymal sperm content was determined using the Triton X100 nuclear solubilization method as described previously [44]. Sperm ultra-structure was assessed using electron microscopy as described previously [45]. Sperm motility was assessed visually using sperm back-flushed from the cauda epididymis [46]. Cauda epididymal sperm morphology was visualized using haematoxylin and eosin staining. The stages of the epithelium tubule were judged by Periodic Acid Schiff (PAS) staining [47]. Apoptosis was evaluated by TUNEL Apoptosis Detection Kit (Millipore) according to the manufacturer’s instructions; n = 5 mice per genotype and the positive cells in 100 seminiferous tubules were counted for each mouse.

Germ and Sertoli cell numbers were counted in 25μm-thick PAS stained methacrylate embedded testis sections using the optical dissector method as described previously [48]. Basal retained elongated spermatids were counted in stage VII-XII [22,48]. N = 5 mice per genotype and 240 counting frames for each mouse.

### Quantitative real time PCR

*Lrguk* in adult tissues and at different time points during the establishment of the first wave of spermatogenesis was defined as outlined previously [49]. *Lrguk* expression was detected using the Taqman assay (Mm01166701-m1). *Lrguk-2* expression was detected using a custom designed assay (Forward primer 5'-CCCCAAAATCTCAAGGTATACTTATCAG-3'; reverse primer 5'–CCGCAGCTGAAGCAAAACTC-3'; probe 5'-CAAAGCACACAATGGT-3'). A custom designed assay was also used to detect both *Lrguk2* and *Lrguk3* transcripts (Forward primer 5'-CTGGCCTATCTGTGGATGACATC-3'; reverse primer 5'-CCGCAGCTGAAGCAAAACTC-3’; probe 5'-CAAAGCACACAATGGT-3’). All expression data was normalized to the *Ppia* house-keeping gene (Mm02342429-gl).

### Germ cell purification and immunochemistry

Germ cells were isolated using the Staput method described previously [50]. For immunofluorescence staining, the purified cells were fixed with 4% paraformaldehyde (PFA) and stained as described previously [9]. Primary antibodies used included: anti-LRGUK (1:200 dilution, Novus Biologicals, raised against amino acids 32–181); anti-HOOK2 (1:200 dilution, GeneTex), anti-acetylated tubulin (1:1000 dilution, Sigma), anti-α-tubulin (1:5000 dilution, Sigma). All primary antibodies were diluted in 10% non-immune horse serum (NHS) in PBS and incubated at 4°C overnight. Secondary antibodies included: Alexa Fluor 555 donkey anti-rabbit IgG and Alexa Fluor 488 donkey anti-mouse IgG, 1:500 dilution in 10% NHS in PBS for 45 minutes at room temperature. Nuclei were labelled with DAPI. The acrosome was visualized using FITC-PNA (1:2000 dilution, Sapphire Bioscience). Images were taken with a SP8 confocal microscope (Leica Mircosystems). For consistence α-tubulin was artificially coloured green in all images.

Paraffin-embedded testis sections were stained for acetylated tubulin as described previously [45].

### Yeast two hybrid screen

The adult mouse testis cDNA library (pDEST22 prey vector) used for yeast two hybrid screen was as described previously [51]. The full length *Lrguk-1* ^*WT*^ and *Lrguk* ^*Kaos*^ cDNAs were cloned into pDEST32 vector using the Gateway cloning kit (Invitrogen). For the initial identification of LRGUK binding partners, the LRGUK^WT^-pDEST32 vector was used as bait in a yeast two hybrid screen with the ProQuest Two-Hybrid System (Invitrogen). Briefly, the LRGUK^WT^-pDEST32 vector and pDEST22 testis cDNA library constructs were co-transformed into Mav203 yeast strain. Putative interacting clones were isolated from the resulting yeast colonies and re-transformed into *E*. *coli* to obtain high purity plasmids for sequencing cDNA inserts. Sequencing of the longest HOOK2 clone revealed an open reading frame of 1078 bp that corresponded to the C-terminal amino acids 369–716 of the HOOK2 protein. To determine the effect of the LRGUK^Kaos^ mutation on the binding to HOOK2, the identified HOOK2-pDEST22 prey vector was co-transformed into the Mav203 yeast strain along with either LRGUK^Kaos^-pDEST32, LRGUK^WT^-pDEST32 or pDEST32 empty vector only and plated on selection media.

### Cloning and co-immunoprecipitation

Full length mouse *Lrguk-1* was amplified from wild type C57BL/6J testis cDNA using *Lrguk-Fw*: 5’-ATATAAAGATCTGCGGCCTTCGAGCGAAAT-3’ and *Lrguk-Rev*: 5’-ATATAAGGGCCCCTATCGCGGCCGTGCGGGAT-3’ primers than cloned into the pEGFP-C1 expression vector (Clontech, Gen Bank Accession number U55763). The LRGUK-1/pEGFP-C1 construct was transfected into HEK293 cells using Lipofectamine 3000 Reagent (Life Technologies, Cat. No. L3000008). Co-immunoprecipitation was carried out using anti-GFP-Trap-A beads as per the manufacturer’s instructions (Chromotek, Cat. No. gta-100). Empty pEGFP-C1 vector was used as a negative control. The presence of LRGUK-GFP and endogenous HOOK2 proteins were determined using anti-GFP (Roche, Cat. No. 11814460001 and HOOK2 antibodies (GeneTex, Cat. No. GTX115898, corresponding with amino acids 197–380 of HOOK2).

### Western blotting

For LRGUK-1 expression, protein was extracted from both wild type and mutant testes using 1% NP-40/PBS lysis buffer with 1:200 protein inhibitor cocktail (Promega). Protein (40μg) was separated on a 12% SDS-PAGE gel. Non-specific antibody binding was minimized by blocking the membrane with 5% skim milk for 1 hour room temperature and antibodies were diluted in 1% skim milk. The membrane was probed with a rabbit LRGUK-1 antibody (Sigma, raised against amino acids 336–427 of LRGUK-1 and thus will not bind to LRGUK-2 or -3). Bound antibody was detected using a goat anti-rabbit IgG HRP (Dako) secondary antibody. Antibody binding was detected using the enhanced chemiluminescence (ECL Plus) detection kit (Amersham Biosciences). Protein loading was normalized against actin.

## Supporting Information

S1 Table The results of a stereological analysis of germ cell content in the *Lrguk* ^*Kaos/Kaos*^ mouse compared to *Lrguk* ^*WT/WT*^ littermates. † Data is expressed as number per Sertoli cell, n = 5 per group, mean±SEM. * denotes p<0.05 compared to *Lrguk* ^*WT/WT*^.
(DOCX)

S1 Fig (A) Body weigh of *Lrguk* ^*WT/WT*^ males versus males (n = 12–13, age 8–12 weeks). (**B-C**) The levels of apoptosis were unchanged in *Lrguk* ^*Kaos/Kaos*^ testes compared to *Lrguk* ^*WT/WT*^. n = 3 per genotype. (D) Stage VIII tubules showing the retention of elongating spermatids in the basal region of the seminiferous tubule in *Lrguk* ^*Kaos/Kaos*^ males.^.^ Note elongating spermatid numbers were also reduced in number and lacked tails. Scale bar = 100μm.
(TIF)

S2 Fig The localization of LRGUK in *Lrguk* ^*WT/WT*^ and *Lrguk* ^*Kaos/Kaos*^ elongating spermatids. The localization of LRGUK (red) in elongating spermatids from *Lrguk* ^*WT/WT*^ and *Lrguk* ^*Kaos/Kaos*^ mice. Scale bar = 5 μm. Manchettes were visualised by α-tubulin (green) and nuclei were stained with DAPI (blue). Manchettes were abnormal in *Lrguk* ^*Kaos/Kaos*^ mice, as indicated by patchy α-tubulin signal. Abnormal manchette movement was indicated by the constriction of the spermatid head. LRGUK immunostaining was more obvious in the marginal ring of *Lrguk* ^*Kaos/Kaos*^ mice (row 3), compared to *Lrguk* ^*WT/WT*^ mice. Please see S2 Fig. for negative control images.
(TIF)

S3 Fig Negative controls of immunofluorescent cell labelling. (**A**) A negative control image for Figs. 3C and 6C- isolated round spermatids. Negative control for secondary antibody donkey anti-rabbit Alexa 488. (**B**) A negative control image for Figs. 3D and 6D (**D**) and S2 Fig. (**F**)—isolated elongating spermatids. Negative control for secondary antibodies donkey anti-rabbit Alexa 488 and donkey anti-mouse Alexa 555. (**C**) A negative control for Figs. 3E and 6E—mouse sperm. Negative control for secondary antibodies donkey anti-rabbit Alexa 488 and donkey anti-mouse Alexa 555. (**E**) A negative control image fro Fig. 5 - isolated elongating spermatids. Negative control for secondary antibody donkey anti-mouse Alexa 555.
(TIF)

## References

[1] McLachlan RI, O'Bryan MK (2010) Clinical Review#: State of the art for genetic testing of infertile men. J Clin Endocrinol Metab 95: 1013–1024. 10.1210/jc.2009-1925 20089613

[2] Cooper TG, Noonan E, von Eckardstein S, Auger J, Baker HW, et al (2010) World Health Organization reference values for human semen characteristics. Hum Reprod Update 16: 231–245. 10.1093/humupd/dmp048 19934213

[3] Baker G, Barak S (2012) Clinical Management of Male Infertility www.ENDOTEXT.org Chapter 7: MDTEXT.COM.Inc, South Dartmouth, MA, USA.

[4] Schultz N, Hamra FK, Garbers DL (2003) A multitude of genes expressed solely in meiotic or postmeiotic spermatogenic cells offers a myriad of contraceptive targets. Proc Natl Acad Sci U S A 100: 12201–12206. 14526100 10.1073/pnas.1635054100 PMC218736

[5] Hermo L, Pelletier RM, Cyr DG, Smith CE (2010) Surfing the wave, cycle, life history, and genes/proteins expressed by testicular germ cells. Part 1: background to spermatogenesis, spermatogonia, and spermatocytes. Microsc Res Tech 73: 241–278. 10.1002/jemt.20783 19941293

[6] Eddy EM (2006) The Spermatozoon In: Neill JD, editor. Knobil and Neill's Physiology of Reproduction. 3 ed: Academic Press pp. 3–54.

[7] Lo JC, Jamsai D, O'Connor AE, Borg C, Clark BJ, et al (2012) RAB-like 2 has an essential role in male fertility, sperm intra-flagellar transport, and tail assembly. PLoS Genet 8: e1002969 10.1371/journal.pgen.1002969 23055941 PMC3464206

[8] Kierszenbaum AL, Rivkin E, Tres LL (2007) Molecular biology of sperm head shaping. Soc Reprod Fertil Suppl 65: 33–43. 17644953

[9] O'Donnell L, Rhodes D, Smith SJ, Merriner DJ, Clark BJ, et al (2012) An essential role for katanin p80 and microtubule severing in male gamete production. PLoS Genet 8: e1002698 10.1371/journal.pgen.1002698 22654669 PMC3359970

[10] Zhou J, Du YR, Qin WH, Hu YG, Huang YN, et al (2009) RIM-BP3 is a manchette-associated protein essential for spermiogenesis. Development 136: 373–382. 10.1242/dev.030858 19091768

[11] de Kretser DM, O’Bryan MK, Lynch M, Reilly A, Kennedy C, et al (2007) The genetics of male infertility. From bench to clinic In: Carrell DT, editor. The Genetics of Male Infertility. Totowa, NJ, USA: Humana Press Inc pp. 251–266.

[12] Hermo L, Pelletier RM, Cyr DG, Smith CE (2010) Surfing the wave, cycle, life history, and genes/proteins expressed by testicular germ cells. Part 2: changes in spermatid organelles associated with development of spermatozoa. Microsc Res Tech 73: 279–319. 10.1002/jemt.20787 19941292

[13] Kierszenbaum AL (2002) Intramanchette transport (IMT): managing the making of the spermatid head, centrosome, and tail. Mol Reprod Dev 63: 1–4. 12211054 10.1002/mrd.10179

[14] Ishikawa H, Marshall WF (2011) Ciliogenesis: building the cell's antenna. Nat Rev Mol Cell Biol 12: 222–234. 10.1038/nrm3085 21427764

[15] Kierszenbaum AL, Rivkin E, Tres LL (2011) Cytoskeletal track selection during cargo transport in spermatids is relevant to male fertility. Spermatogenesis 1: 221–230. 22319670 10.4161/spmg.1.3.18018 PMC3271664

[16] Chemes HE (2012) Sperm Centrioles and Their Dual Role in Flagellogenesis and Cell Cycle of the Zygote In: Schatten H, editor. The Centrosome: Humana Press pp. 33–48.

[17] Kierszenbaum AL, Tres LL, Rivkin E, Kang-Decker N, van Deursen JM (2004) The acroplaxome is the docking site of Golgi-derived myosin Va/Rab27a/b- containing proacrosomal vesicles in wild-type and Hrb mutant mouse spermatids. Biol Reprod 70: 1400–1410. 14724135 10.1095/biolreprod.103.025346

[18] Kierszenbaum AL, Tres LL (2004) The acrosome-acroplaxome-manchette complex and the shaping of the spermatid head. Arch Histol Cytol 67: 271–284. 15700535 10.1679/aohc.67.271

[19] Baron Gaillard CL, Pallesi-Pocachard E, Massey-Harroche D, Richard F, Arsanto JP, et al (2011) Hook2 is involved in the morphogenesis of the primary cilium. Mol Biol Cell 22: 4549–4562. 10.1091/mbc.E11-05-0405 21998199 PMC3226474

[20] Szebenyi G, Hall B, Yu R, Hashim AI, Kramer H (2007) Hook2 localizes to the centrosome, binds directly to centriolin/CEP110 and contributes to centrosomal function. Traffic 8: 32–46. 17140400 10.1111/j.1600-0854.2006.00511.x

[21] Jamsai D, O'Bryan MK (2010) Genome-wide ENU mutagenesis for the discovery of novel male fertility regulators. Syst Biol Reprod Med 56: 246–259. 10.3109/19396361003706424 20536324

[22] O'Donnell L, Nicholls PK, O'Bryan MK, McLachlan RI, Stanton PG (2011) Spermiation: The process of sperm release. Spermatogenesis 1: 14–35. 21866274 10.4161/spmg.1.1.14525 PMC3158646

[23] Kleene KC (1996) Patterns of translational regulation in the mammalian testis. Mol Reprod Dev 43: 268–281. 8824926 10.1002/(SICI)1098-2795(199602)43:2<268::AID-MRD17>3.0.CO;2-#

[24] Berruti G, Paiardi C (2011) Acrosome biogenesis: Revisiting old questions to yield new insights. Spermatogenesis 1: 95–98. 22319656 10.4161/spmg.1.2.16820 PMC3271650

[25] Yang WX, Sperry AO (2003) C-terminal kinesin motor KIFC1 participates in acrosome biogenesis and vesicle transport. Biol Reprod 69: 1719–1729. 12826589 10.1095/biolreprod.102.014878

[26] Reese ML, Dakoji S, Bredt DS, Dotsch V (2007) The guanylate kinase domain of the MAGUK PSD-95 binds dynamically to a conserved motif in MAP1a. Nat Struct Mol Biol 14: 155–163. 17220895 10.1038/nsmb1195

[27] Kobe B, Kajava AV (2001) The leucine-rich repeat as a protein recognition motif. Curr Opin Struct Biol 11: 725–732. 11751054 10.1016/s0959-440x(01)00266-4

[28] Linstedt AD (2004) Positioning the Golgi apparatus. Cell 118: 271–272. 15294150 10.1016/j.cell.2004.07.015

[29] Singla V, Reiter JF (2006) The primary cilium as the cell's antenna: signaling at a sensory organelle. Science 313: 629–633. 16888132 10.1126/science.1124534

[30] Paintrand M, Moudjou M, Delacroix H, Bornens M (1992) Centrosome organization and centriole architecture: their sensitivity to divalent cations. J Struct Biol 108: 107–128. 1486002 10.1016/1047-8477(92)90011-x

[31] Tanos BE, Yang HJ, Soni R, Wang WJ, Macaluso FP, et al (2013) Centriole distal appendages promote membrane docking, leading to cilia initiation. Genes Dev 27: 163–168. 10.1101/gad.207043.112 23348840 PMC3566309

[32] Delgehyr N, Sillibourne J, Bornens M (2005) Microtubule nucleation and anchoring at the centrosome are independent processes linked by ninein function. J Cell Sci 118: 1565–1575. 15784680 10.1242/jcs.02302

[33] Mogensen MM, Malik A, Piel M, Bouckson-Castaing V, Bornens M (2000) Microtubule minus-end anchorage at centrosomal and non-centrosomal sites: the role of ninein. J Cell Sci 113 (Pt 17): 3013–3023. 10934040 10.1242/jcs.113.17.3013

[34] Moynihan KL, Pooley R, Miller PM, Kaverina I, Bader DM (2009) Murine CENP-F regulates centrosomal microtubule nucleation and interacts with Hook2 at the centrosome. Mol Biol Cell 20: 4790–4803. 10.1091/mbc.E09-07-0560 19793914 PMC2777108

[35] Braun RE (1998) Post-transcriptional control of gene expression during spermatogenesis. Semin Cell Dev Biol 9: 483–489. 9813196 10.1006/scdb.1998.0226

[36] Gadella BM (2012) Dynamic regulation of sperm interactions with the zona pellucida prior to and after fertilisation. Reprod Fertil Dev 25: 26–37. 10.1071/RD12277 23244826

[37] Kerr J, Loveland KL, O’Bryan MK, de Kretser DM (2006) The Cytology of the Testis and Intrinsic Control Mechanisms In: Neill JD, Challis JRG, de Kretser DM, Pfaff DW, Richards JS et al, editors. The Physiology of Reproduction. 3rd ed. St. Louis, MO: Elsevier Academic Press pp. 827–947.

[38] Mendoza-Lujambio I, Burfeind P, Dixkens C, Meinhardt A, Hoyer-Fender S, et al (2002) The Hook1 gene is non-functional in the abnormal spermatozoon head shape (azh) mutant mouse. Hum Mol Genet 11: 1647–1658. 12075009 10.1093/hmg/11.14.1647

[39] Xu L, Sowa ME, Chen J, Li X, Gygi SP, et al (2008) An FTS/Hook/p107(FHIP) complex interacts with and promotes endosomal clustering by the homotypic vacuolar protein sorting complex. Mol Biol Cell 19: 5059–5071. 10.1091/mbc.E08-05-0473 18799622 PMC2592673

[40] Davis EE, Katsanis N (2012) The ciliopathies: a transitional model into systems biology of human genetic disease. Curr Opin Genet Dev 22: 290–303. 10.1016/j.gde.2012.04.006 22632799 PMC3509787

[41] Lehti MS, Kotaja N, Sironen A (2013) KIF3A is essential for sperm tail formation and manchette function. Mol Cell Endocrinol 377: 44–55. 10.1016/j.mce.2013.06.030 23831641

[42] Taulman PD, Haycraft CJ, Balkovetz DF, Yoder BK (2001) Polaris, a protein involved in left-right axis patterning, localizes to basal bodies and cilia. Mol Biol Cell 12: 589–599. 11251073 10.1091/mbc.12.3.589 PMC30966

[43] Borg CL, Wolski KM, Gibbs GM, O'Bryan MK (2010) Phenotyping male infertility in the mouse: how to get the most out of a 'non-performer'. Hum Reprod Update 16: 205–224. 10.1093/humupd/dmp032 19758979 PMC2816191

[44] Cotton L, Gibbs GM, Sanchez-Partida LG, Morrison JR, de Kretser DM, et al (2006) FGFR-1 [corrected] signaling is involved in spermiogenesis and sperm capacitation. J Cell Sci 119: 75–84. 16352663 10.1242/jcs.02704

[45] Arsov T, Silva DG, O'Bryan MK, Sainsbury A, Lee NJ, et al (2006) Fat aussie—a new Alstrom syndrome mouse showing a critical role for ALMS1 in obesity, diabetes, and spermatogenesis. Mol Endocrinol 20: 1610–1622. 16513793 10.1210/me.2005-0494

[46] Gibbs GM, Orta G, Reddy T, Koppers AJ, Martinez-Lopez P, et al (2011) Cysteine-rich secretory protein 4 is an inhibitor of transient receptor potential M8 with a role in establishing sperm function. Proc Natl Acad Sci U S A 108: 7034–7039. 10.1073/pnas.1015935108 21482758 PMC3084142

[47] Ahmed EA, de Rooij DG (2009) Staging of mouse seminiferous tubule cross-sections. Methods Mol Biol 558: 263–277. 10.1007/978-1-60761-103-5_16 19685330

[48] Saito K, O'Donnell L, McLachlan RI, Robertson DM (2000) Spermiation failure is a major contributor to early spermatogenic suppression caused by hormone withdrawal in adult rats. Endocrinology 141: 2779–2785. 10919263 10.1210/endo.141.8.7628

[49] Vergouwen RP, Huiskamp R, Bas RJ, Roepers-Gajadien HL, Davids JA, et al (1993) Postnatal development of testicular cell populations in mice. J Reprod Fertil 99: 479–485. 8107030 10.1530/jrf.0.0990479

[50] Romrell LJ, Bellve AR, Fawcett DW (1976) Separation of mouse spermatogenic cells by sedimentation velocity. A morphological characterization. Dev Biol 49: 119–131. 176074 10.1016/0012-1606(76)90262-1

[51] Ly-Huynh JD, Lieu KG, Major AT, Whiley PA, Holt JE, et al (2011) Importin alpha2-interacting proteins with nuclear roles during mammalian spermatogenesis. Biol Reprod 85: 1191–1202. 10.1095/biolreprod.111.091686 21900684

