# Supplementary material for: LRGUK-1 Is Required for Basal Body and Manchette Function during Spermatogenesis and Male Fertility
Source: PLoS Genet. 2015 Mar 17;11(3):e1005090. doi: 10.1371/journal.pgen.1005090 (PMC4363142; doi:10.1371/journal.pgen.1005090)
Supplement: S1 Table — † Data is expressed as number per Sertoli cell, n = 5 per group, mean±SEM. * denotes p<0.05 compared to Lrguk WT/WT. (DOCX) [file pgen.1005090.s001.docx]

**S1 Table.**

|  | **Pachytene spermatocytes** | **Round spermatids**  **Steps 1-8** | **Elongating spermatids**  **Steps 9-12** | **Elongated spermatids**  **Steps 13-16** |
| --- | --- | --- | --- | --- |
| WT† | 4.02±0.33 | 10.15±1.11 | 2.89±0.35 | 9.51±0.69 |
| Mutant† | 3.81±0.10 | 10.19±0.55 | 3.10±0.24 | 6.00±0.68 * |
