# Supplementary figures and images for: LRGUK-1 Is Required for Basal Body and Manchette Function during Spermatogenesis and Male Fertility
Source: PLoS Genet. 2015 Mar 17;11(3):e1005090. doi: 10.1371/journal.pgen.1005090 (PMC4363142; doi:10.1371/journal.pgen.1005090)

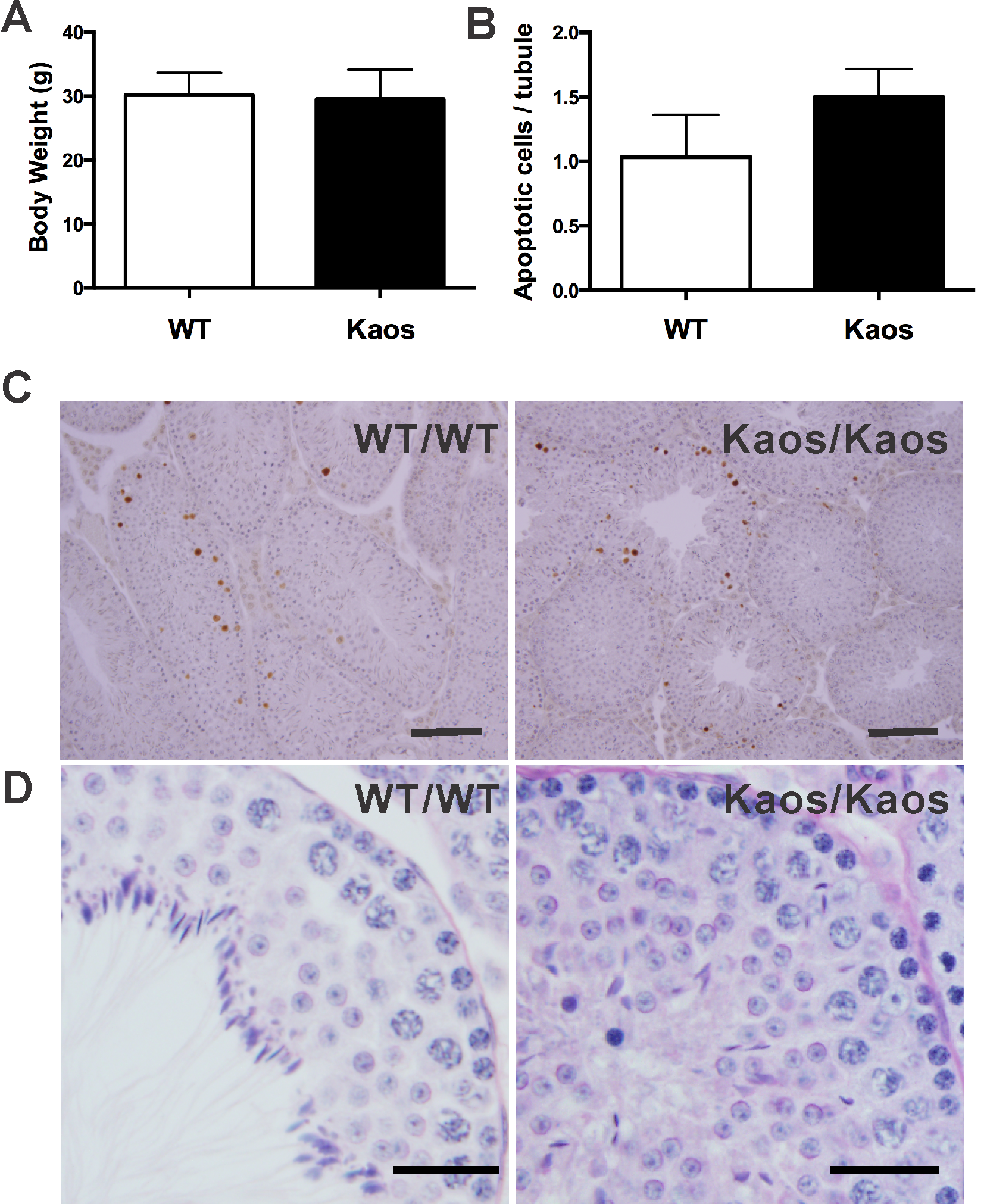

Supplement: S1 Fig — (B-C) The levels of apoptosis were unchanged in Lrguk Kaos/Kaos testes compared to Lrguk WT/WT. n = 3 per genotype. (D) Stage VIII tubules showing the retention of elongating spermatids in the basal region of the seminiferous tubule in Lrguk Kaos/Kaos males.. Note elongating spermatid numbers were also reduced in number and lacked tails. Scale bar = 100μm. (TIF) [file pgen.1005090.s002.tif]

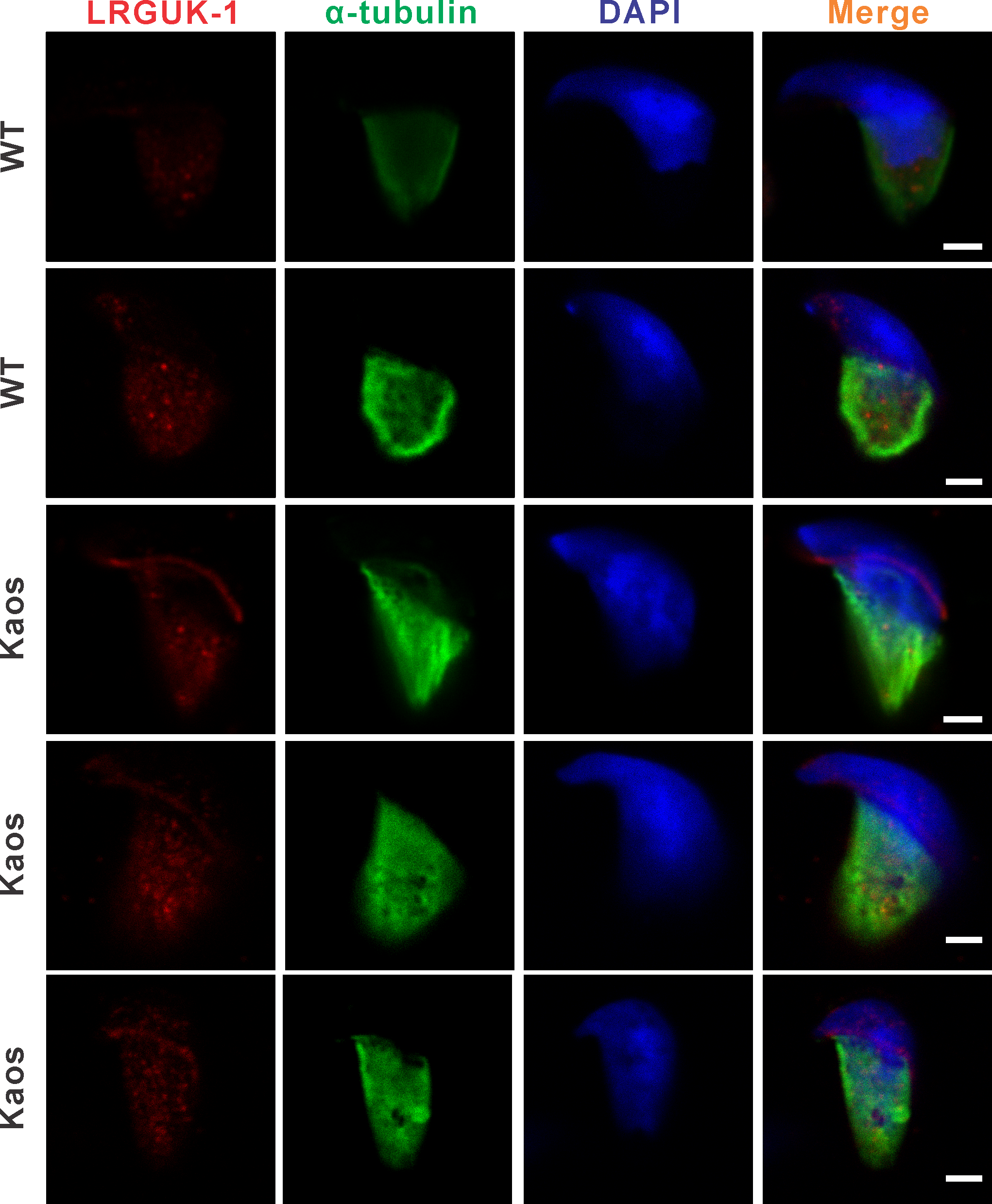

Supplement: S2 Fig — The localization of LRGUK (red) in elongating spermatids from Lrguk WT/WT and Lrguk Kaos/Kaos mice. Scale bar = 5 μm. Manchettes were visualised by α-tubulin (green) and nuclei were stained with DAPI (blue). Manchettes were abnormal in Lrguk Kaos/Kaos mice, as indicated by patchy α-tubulin signal. Abnormal manchette movement was indicated by the constriction of the spermatid head. LRGUK immunostaining was more obvious in the marginal ring of Lrguk Kaos/Kaos mice (row 3), compared to Lrguk WT/WT mice. Please see S2 Fig. for negative control images. (TIF) [file pgen.1005090.s003.tif]

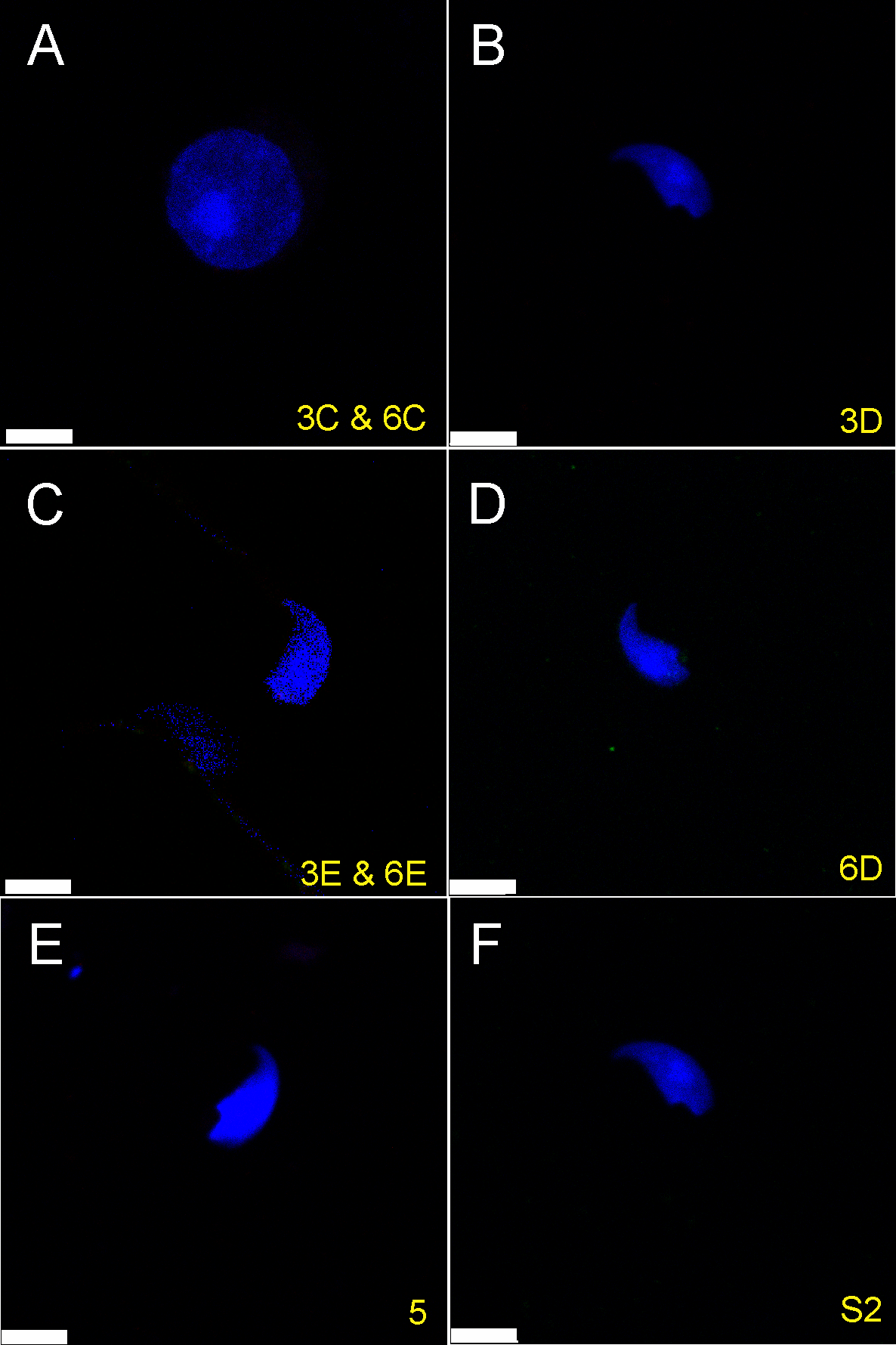

Supplement: S3 Fig — (A) A negative control image for Figs. 3C and 6C- isolated round spermatids. Negative control for secondary antibody donkey anti-rabbit Alexa 488. (B) A negative control image for Figs. 3D and 6D (D) and S2 Fig. (F)—isolated elongating spermatids. Negative control for secondary antibodies donkey anti-rabbit Alexa 488 and donkey anti-mouse Alexa 555. (C) A negative control for Figs. 3E and 6E—mouse sperm. Negative control for secondary antibodies donkey anti-rabbit Alexa 488 and donkey anti-mouse Alexa 555. (E) A negative control image fro Fig. 5 - isolated elongating spermatids. Negative control for secondary antibody donkey anti-mouse Alexa 555. (TIF) [file pgen.1005090.s004.tif]
